# A miR-200b/200c/429-Binding Site Polymorphism in the 3′ Untranslated Region of the AP-2α Gene Is Associated with Cisplatin Resistance

**DOI:** 10.1371/journal.pone.0029043

**Published:** 2011-12-14

**Authors:** Yuan Wu, Yuzhong Xiao, Xiaofeng Ding, Yiming Zhuo, Peng Ren, Chang Zhou, Jianlin Zhou

**Affiliations:** Key Laboratory of Protein Chemistry and Developmental Biology of Ministry of Education, College of Life Science, Hunan Normal University, Changsha, China; The University of Arizona, United States of America

## Abstract

The transcription factor AP-2α functions as a tumor suppressor by regulating various genes that are involved in cell proliferation and apoptosis. Chemotherapeutic drugs including cisplatin induce post-transcriptionally endogenous AP-2α, which contributes to chemosensitivity by enhancing therapy-induced apoptosis. microRNAs (miRNAs) miR-200b, miR-200c and miR-429 (miR-200b/200c/429) are up-regulated in endometrial and esophageal cancers, and their overexpression correlates with resistance to cisplatin treatment. Using computational programs, we predicted that the 3′ untranslated region (UTR) of AP-2α gene contains a potential miRNA response element (MRE) for the miR-200b/200c/429 family, and the single nucleotide polymorphism (SNP) site rs1045385 (A or C allele) resided within the predicted MRE. Luciferase assays and Western blot analysis demonstrated that the miR-200b/200c/429 family recognized the MRE in the 3′ UTR of AP-2α gene and negatively regulated the expression of endogenous AP-2α proteins. SNP rs1045385 A>C variation enhanced AP-2α expression by disrupting the binding of the miR-200b/200c/429 family to the 3′ UTR of AP-2α. The effects of the two polymorphic variants on cisplatin sensitivity were determined by clonogenic assay. The overexpression of AP-2α with mutant 3′ UTR (C allele) in the endometrial cancer cell line HEC-1A, which has high levels of endogenous miR-200b/200c/429 and low levels of AP-2α protein, significantly increased cisplatin sensitivity, but overexpression of A allele of AP-2α has no significant effects, compared with mock transfection. We concluded that miR-200b/200c/429 induced cisplatin resistance by repressing AP-2α expression in endometrial cancer cells. The SNP (rs1045385) A>C variation decreased the binding of miR-200b/200c/429 to the 3′ UTR of AP-2α, which upregulated AP-2α protein expression and increased cisplatin sensitivity. Our results suggest that SNP (rs1045385) may be a potential prognostic marker for cisplatin treatment.

## Introduction

The AP-2 family of transcription factors is involved in the regulation of embryonic development, cell proliferation and tumorigenesis. To date, five members of the AP-2 family have been identified: AP-2α, AP-2β, AP-2γ, AP-2δ and AP-2ε. All AP-2 proteins bind as homo- or heterodimers to the consensus sequence of 5′- GCCNNNGGC-3′ and directly regulate transcription of their target genes (reviewed in [1]). Among these, AP-2α is the best-characterized gene. The importance of AP-2α during embryogenesis has been demonstrated using knock-out mice studies. Loss of AP-2α leads to defects in the neural tube, face, eyes, heart, body wall and limbs [2], [3], [4]. In addition to its roles in embryonic development, AP-2α functions as a tumor suppressor by regulating the transcription of various genes that are involved in cell proliferation and apoptosis. AP-2α regulates the transcriptional activation of p21WAF1/CIP1[5], E-cadherin[6] and PTEN[7] and transcriptional repression of Bcl-2[8], vascular endothelial growth factor[9] and mucin MUC4 [10]. AP-2α expression is down-regulated in skin, brain, breast, ovarian and colon cancers, and its lower expression predicts poor survival of patients[11], [12], [13]. Moreover, several studies have shown that AP-2α status is associated with the chemosensitivity of cancer cells[14], [15], [16]. Endogenous AP-2α protein is posttranscriptionally induced by various chemotherapeutic drugs, including cisplatin, adriamycin and taxol, and promotes chemosensitivity by enhancing therapy-induced apoptosis in colon and breast cancer cells [14]. A moderate overexpression of AP-2α in pancreatic cancer cell line CAPAN-1 increased the chemosensitivity to low doses of gemcitabine[15]. The expression of AP-2α in the lung carcinoma cell line H460 increased the chemosensitivity to adriamycin (2.5-fold) and cisplatin (5-fold)[16].

Recently, microRNAs (miRNAs) have attracted more attention because they have regulatory roles in a broad range of biological processes, including embryogenesis, differentiation, proliferation and apoptosis, as well as in carcinogenesis. The miRNAs are a class of small (approximately 22 nucleotides), single-stranded, endogenous non-coding RNAs that negatively regulate gene expression by binding to the 3′ untranslated region (UTR) of target mRNA to inhibit translation and/or promote mRNA degradation (reviewed in [17], [18]). Approximately 30% of human genes are regulated by miRNAs[19]. Therefore, we proposed that miRNA might also regulate AP-2α. Using several computational programs, we identified a potential binding site (miRNA response element, MRE) of miR-200b, miR-200c and miR-429 (miR-200b/200c/429) in the 3′ UTR of the AP-2α gene. The miRNAs miR-200b/200c/429 share a common seed sequence of AAUACUG[20] and are highly expressed in endometrial cancers than in normal endometrial tissues[21]. In this report, we demonstrated that AP-2α was directly regulated by miR-200b/200c/429 family and that the single nucleotide polymorphism (SNP) rs1045385 was located in the miR-200b/200c/429-binding site of the 3′ UTR of AP-2α and affected AP-2α protein expression and cisplatin resistance in endometrial cancer cells.

## Results

### The 3′ UTR of AP-2α gene contains a MRE for miR-200b/200c/429 family

**Figure 1:**
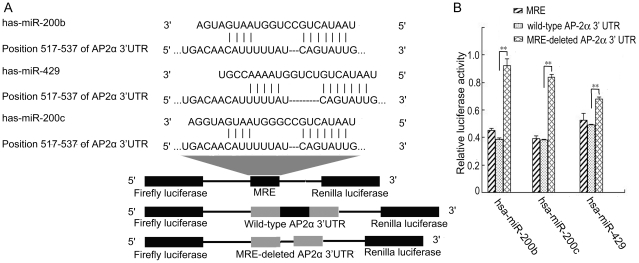
The 3′ UTR of AP-2α contains a MRE for miR-200b/200c/429 family. A) Diagram of luciferase reporter constructs. The predicted MRE, wild-type or MRE-deleted AP-2α 3′ UTRs were inserted downstream of the firefly luciferase gene of the pmirGLO vector. B) Repression of firefly luciferase by the interaction between miRNA and the predicted MRE. Each luciferase construct was co-transfected with miR-200b, miR-200c or miR-429 mimics into HEK293 cells. At 24 h post-transfection, the luciferase activity was examined. The firefly luciferase activity was normalized to Renilla luciferase activity. The firefly luciferase activity of the cells that were transfected with miRNA mimics was represented as the percent activity relative to that of the cells that were transfected with negative control miRNA mimics. Data are shown as the mean±SD of three independent experiments. **, p<0.01.

To identify the miRNAs that regulated AP-2α, we used four computational programs (TargetScan, Microcosm, DIANA-microT and miRanda) to search for MREs in the 3′-UTR of the AP-2α gene. Each program predicted different MREs. However, the programs identified a common MRE for the miR-200b/200c/429 family (Fig. 1a). Moreover, this putative MRE was highly conserved in vertebrates (data not shown).

We validated whether the predicted MRE could be recognized by the miR-200b/200c/miR-429 family using the dual-luciferase vector pmirGLO. The predicted MRE, wild-type or MRE-deleted 3′ UTR of AP-2α was cloned downstream of the firefly luciferase of the pmirGLO vector and co-transfected with miR-200b, miR-200c or miR-429 mimics (double-stranded processed miRNA) into HEK293 cells, which do not express miR-200b, miR-200c or miR-429[21]. As shown in Fig.1b, the expression of miR-200b, miR-200c or miR-429 suppressed the firefly luciferase activities of MRE and the MRE-containing 3′ UTR of AP-2α. However, the luciferase activity was restored using MRE-deleted 3′ UTR of AP-2α. These results indicate that the predicted MRE mediates the binding of the miR-200b/200c/429 family to AP-2α.

### The miR-200b/200c/429 family represses endogenous AP-2α expression

**Figure 2:**
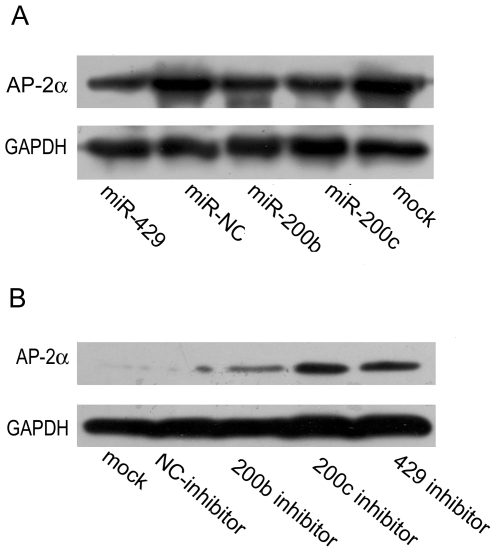
The miR-200b/200c/429 family represses endogenous AP-2α expression. The miRNA mimics and inhibitors were transfected into HeLa cells (A) and HEC-1A cells (B), respectively. At 36 h after transfection, cells lysates were prepared and subjected to Western blot analysis.

To examine the effect of the miR-200b/200c/429 family on endogenous AP-2α expression, we transfected miR-200b, miR-200c or miR-429 mimics into cervical cancer HeLa cells, which is known to express high levels of AP-2α protein[22]. Enhanced expression of miR-200b, miR-200c or miR-429 in HeLa cells significantly decreased the amount of AP-2α protein, compared with mock transfection (Fig. 2a).

To further confirm these results, we blocked the expression of miR-200b, miR-200c or miR-429 using miRNA inhibitors (single-stranded complementary miRNA) in the endometrial cancer cell line HEC-1A, which has been shown to express higher levels of miR-200b, miR-200c or miR-429[21]. Cells that were untreated or transfected with a negative control expressed low levels of AP-2α. However, the silencing of miR-200b, miR-200c and miR-429 significantly increased AP-2α levels, compared with mock transfection (Fig. 2b).

These results demonstrate that miR-200b, 200c and miR-429 negatively regulate AP-2α expression.

### SNP rs1045385 A>C variation enhances AP-2α expression by interfering with the miR-200b/200c/429 family

**Figure 3:**
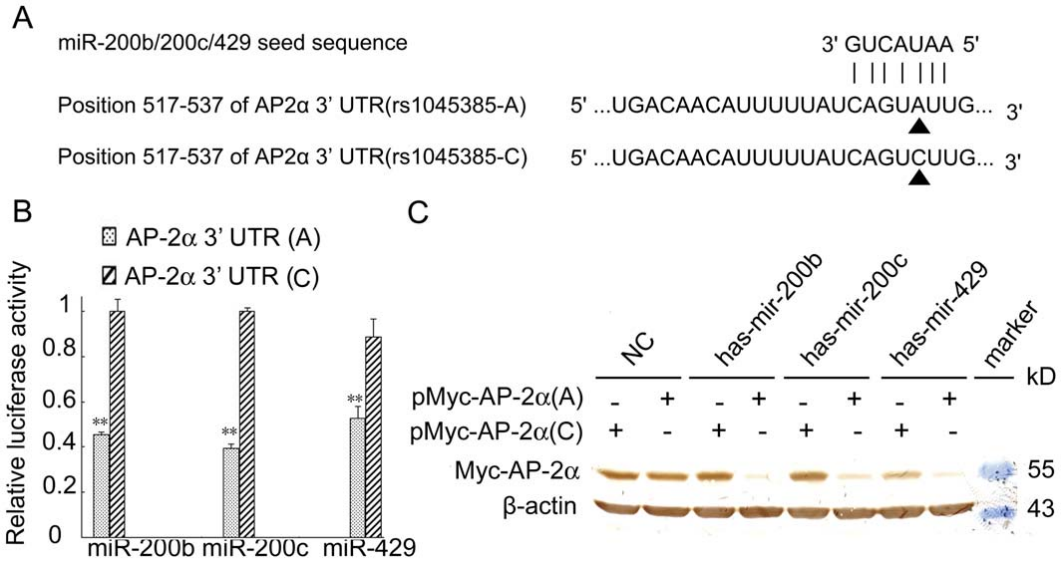
SNP rs1045385 A>C variation in the AP-2α 3′ UTR inhibits the binding of the miR-200b/200c/429 family and enhances AP-2α expression. A) Diagram of the binding between miR-200b/200c/429 seed sequence and the AP-2α 3′ UTR with the A or C allele. B) SNP rs1045385 A>C variation in the AP-2α 3′ UTR inhibited the binding of the miR-200b/200c/429 family. HEK293 cells were transfected with indicated miRNA mimics and luciferase reporter constructs containing the AP-2α 3′ UTR with the A or C allele. At 24 h post-transfection, luciferase activity was examined. The firefly luciferase activity was normalized to the Renilla luciferase activity. The firefly luciferase activity of the cells that were transfected with miRNA mimics was represented as the percent activity relative to that of the cells that were transfected with negative control miRNA mimics. **, p<0.01. C) SNP rs1045385 A>C variation in the AP-2α 3′ UTR enhanced the AP-2α expression in HEC-1A cells. The Myc-tagged expression construct of full-length AP-2α with the A or C allele was co-transfected with miRNA mimics into HEC-1A cells. At 36 h after transfection, cell lysates were prepared and subjected to Western blot analysis.

By searching the Ensembl database, we identified a SNP (rs1045385) in the miR-200b/200c/429-binding MRE of the AP-2α 3′ UTR. The SNP rs1045385 has two alleles, A and C alleles (Fig. 3a). To investigate the effect of SNP rs1045385 A>C variation on miRNA binding, the wild-type and mutant 3′ UTR were cloned into the dual-luciferase reporter vector pmirGLO and co-transfected with miRNA mimics into HEK293 cells. As shown in Fig. 3b, A>C substitution in MRE increased the luciferase activity of the AP-2α 3′ UTR, indicating A>C substitution suppressed the binding of miR-200b/200c/miR-429 to their target.

To determine the effect of SNP rs1045385 on miR-200b/200c/miR-429-mediated regulation of AP-2α, we generated expression constructs of AP-2α containing a wild-type 3′ UTR or a mutant 3′ UTR, i.e., pMyc-AP-2α (A) and pMyc-AP-2α (C), respectively, and co-transfected them with miR-200b, miR-200c or miR-429 mimics into HEK293 cells. The overexpression of miR-200b or miR-200c decreased the expression of the AP-2α gene with a wild-type 3′ UTR but had no effect on that of the AP-2α gene with a mutant 3′ UTR (Fig. 3c). Although miR-429 transfection suppressed the expression of wild-type and mutant AP-2α, the cells transfected with the mutant AP-2α gene had higher AP-2α protein levels than those transfected with the wild-type AP-2α gene. The expression of wild-type and MRE-mutated AP-2α was suppressed by miR-429, suggesting that the 3′ UTR of AP-2α contained another binding site for miR-429.

Taken together, these results indicate that SNP rs1045385 A>C variation mediates AP-2α upregulation by disrupting the binding of miR-200b/200c/429 to the 3′ UTR of AP-2α.

### SNP rs1045385 A>C variation increases cisplatin sensitivity of HEC-1A cells

**Figure 4:**
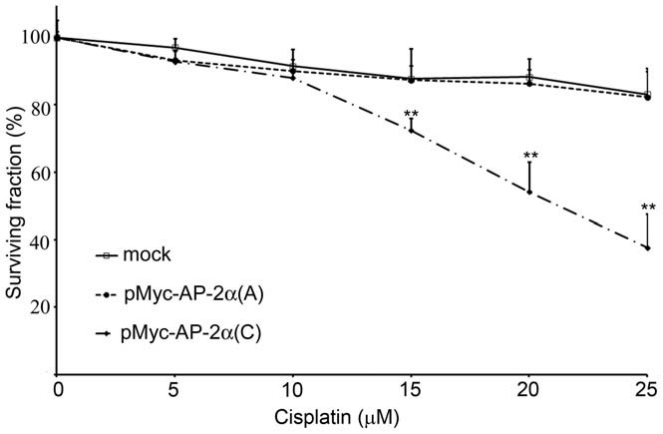
SNP rs1045385 A>C variation increases cisplatin sensitivity of HEC-1A cells. The Myc-tagged expression constructs of full-length AP-2α with the A or C allele were transfected into HEC-1A cells. At 12 h after transfection, the cells were plated in 6-well plates, and treated with the indicated concentration of cisplatin for 6 h. The cell viability was determined using the clonogenic assay. Data are shown as the mean±SD of three independent experiments. **, p<0.01.

As stated above, SNP rs1045385 A>C variation enhanced the expression of AP-2α protein, while AP-2α overexpression has been shown to promote chemosensitivity in tumor cells[14], [15], [16]. Therefore, we tested whether SNP rs1045385 affected the response to cisplatin treatment. HEC-1A cells were transfected with wild-type (A allele) or MRE-mutated (C allele) AP-2α cDNA constructs, and treated with various concentrations of cisplatin for 6 h. The result of the clonogenic assay showed that the transfection of C allele of AP-2α significantly inhibited cell viability, but overexpression of A allele of AP-2α has no significant effects, compared with mock transfection. At concentrations higher than 10 µM, the survival fraction of the cells that were transfected with mutant AP-2α was significantly lower than that of the cells that were transfected with wild-type AP-2α (Fig. 4). As stated above, the mutant AP-2α was insensitive to miR-200b/200c/429. Therefore, cells with mutant AP-2α displayed higher levels of AP-2α protein in the HEC-1A cells containing higher levels of miR-200b/200c/429 than those with wild-type AP-2α, which exhibited increased cisplatin sensitivity. These results indicate that SNP rs1045385 A>C variation increases cisplatin sensitivity in HEC-1A cells.

## Discussion

Here, we present evidence that miR-200b, miR-200c and miR-429 are negative regulators of AP-2α. Using computational programs, we predicted that the 3′ UTR of AP-2α contained a potential binding site for miR-200b, miR-200c and miR-429. We validated the binding of miR-200b/200c/429 to the 3′ UTR of AP-2α using the luciferase assay. Finally, we examined the effects of miR-200b, miR-200c and miR-429 on the expression of endogenous AP-2α. Ectopic expression of miR-200b, miR-200c or miR-429 suppressed the expression of AP-2α in HeLa cells, whereas inhibition of miR-200b, miR-200c or miR-429 in HEC-1A enhanced the expression of AP-2α. To our knowledge, this is the first report that has identified miRNAs that directly regulate AP-2α.

Enhanced expression of AP-2α in cancer cells increase the sensitivity to cisplatin[14], [16]. The miR-200 family is highly expressed in endometrial cancers than in normal endometrial tissues, and its overexpression is correlated with cisplatin resistance [21], [23]. Lee et al. have reported that specific inhibition using anti-miR-429 enhanced cisplatin-induced cytoxicity in HEC-1 cells. In this study, we showed that AP-2α overexpression in endometrial cancer HEC-1 cells also increased the sensitivity to cisplatin treatment. The cells that were transfected with mutant AP-2α, which contains a mutated 3′ UTR that cannot bind to miR-200b/200b/429, were more sensitive to cisplatin than the cells with wild-type AP-2α. These results suggest that miR-200b/miR-200c/miR-429 overexpression induces cisplatin resistance by repressing AP-2α expression in HEC-1 cells.

SNPs are the most common type of genetic variation in human genomes. When SNPs are located at or near a MRE of a functional gene, they may affect gene expression by altering the interaction between miRNA and mRNA [24], [25]. Increasing evidence has suggested that MRE polymorphisms are associated with tumor susceptibility and chemotherapeutic response[24], [26], [27]. For example, a miR-4-binding site polymorphism in the dihydrofolate reductase gene leads to methotrexate resistance[26]. Therefore, identifying SNPs that are associated with cancer and chemosensitivity is valuable for personalized cancer diagnostic and therapeutic approaches. Several studies have shown that AP-2α status is associated with the chemosensitivity of cancer cells[14], [15], [16] Here, we found that the SNP (rs1045385) A>C variation in the AP-2α 3′ UTR disrupted the interaction between the miR-200b/200c/429 family and AP-2α, which upregulated AP-2α expression and AP-2α-mediated cisplatin sensitivity. Our results suggest that SNP rs1045385 may be a potential prognostic marker for cisplatin treatment and that patients with the C allele of SNP rs1045385 may be more sensitive to chemotherapy than those with the wild-type A allele of SNP rs1045385.

## Materials and Methods

### Computational analysis of MRE

The 3′ UTR of the AP-2α gene was obtained from the Ensembl database (http://www.ensembl.org). The following computational programs were used to search for potential MREs in the 3′ UTR of the AP-2α gene: TargetScan[19] (http://www.targetscan.org/), MicroCosm Targets (http://www.ebi.ac.uk/enright-srv/microcosm), DIANA-microT[28] (http://diana.cslab.ece.ntua.gr/microT) and miRanda[29] (http://www.microrna.org).

### Construction and mutagenesis of dual-luciferase reporter plasmids and AP-2α expression plasmids

The 3′ UTR of AP-2α was amplified from HeLa cDNA using a nested PCR and was inserted into the 3′-end of the firefly luciferase gene of the dual-luciferase miRNA target expression vector pmirGLO (Promega Corporation, Madison, WI, USA) between Pmel and XbaI sites. The oligonucleotide pairs that contain the MRE of the desired miRNA were designed to form overhangs that were complementary to those generated by the annealed fragments of PmeI and XbaI digestion. The oligonucleotide pairs were synthesized, annealed and ligated into the pmirGLO vector. The full AP-2α cDNA construct containing the entire 3′ UTR was constructed by inserting the 3′ UTR of AP-2α into a Myc-tagged AP-2α expression plasmid. Deletion and site-directed mutagenesis were performed by overlapping PCR as described previously[30].

### Cell culture and transfection

Human embryonic kidney-293 (HEK293) cells, cervical cancer HeLa cells and endometrial cancer HEC-1A cells (from American Tissue Culture Collection, ATCC, Manassas, VA,USA) were cultured in DMEM or McCoy's 5a media that was su\lemented with glutamine, antibiotics and 10% fetal bovine serum at 37°C and 5% CO_2_. Transfection was performed using Lipofectamine 2000 (Invitrogen, Carlsbad, CA, USA) according to the manufacturer's instructions.

### Luciferase assay

The dual-luciferase reporter plasmids were co-transfected with miRNA mimics (GenePharma, Shanghai, China) into HEK293 cells. At 24 h post-transfection, cells were assayed for luciferase activity using the Dual-Glo Luciferase Assay System (Promega) according to the manufacturer's instructions. The firefly luciferase activities were normalized to Renilla luciferase activity. The firefly luciferase activity of the cells that were transfected with miRNA mimics was represented as the percent activity relative to that of the cells that were transfected with negative control miRNA mimics. For each transfection, the luciferase activity was averaged from three replicates.

### Western blot analysis to assess the effects of miRNA mimics and inhibitors on AP-2α expression

All of the miRNA mimics and inhibitors that were used in this study were purchased from GenePharma (Shanghai, China). At 36 h posttransfection, cells were harvested and lysed in RIPA buffer [50 mM Tris–HCl (pH 7.2), 150 mM NaCl, 1% (v/v) Triton X-100, 1% (w/v) sodium deoxycholate, 0.1% (w/v) SDS] with protease inhibitors. Proteins were separated on 10% SDS-polyacrylamide gel and transferred to PVDF membranes. A PageRuler prestained protein ladder was used as a molecular marker. A single membrane was cut into two parts at the 40-kD band and incubated with anti-AP-2α (Abcam, Cambridge, UK) and anti-GAPDH (Santa Cruz, CA, USA) primary antibodies, respectively. The protein was detected using a HRP-conjugated secondary antibody and a ChemiLucent ECL Detection System (Millipore, Billerica, MA, USA).

### Cisplatin treatment and clonogenic assays

The clonogenic assay was performed as described by Franken et al[31]. HEC-1 cells were transfected with expression constructs of AP-2α containing a wild-type or mutant 3′ UTR. At 12 h posttransfection, the cells were plated in 6-well plates, and treated with various concentrations (0, 5, 10,15,20,25 µmol/L) of cisplatin (Sigma-Aldrich) for 6 h. After treatment, cells were maintained in fresh media for 2 weeks. Colonies were fixed with methanol and stained with Giemsas for 20 min. The cell viability was analyzed by the surviving fraction[31].

### Statistical analysis

Microsoft Excel was used for statistical analysis. Student's t-test was performed to evaluate the significance of difference between samples.
